# Effects of Gamification on Behavioral Change in Education: A Meta-Analysis

**DOI:** 10.3390/ijerph18073550

**Published:** 2021-03-29

**Authors:** Jihoon Kim, Darla M. Castelli

**Affiliations:** 1 Physical Education Teacher Education, Department of Curriculum and Instruction, The University of Texas at Austin, Austin, TX 78712, USA; jihoonkim@utexas.edu; 2 Health Behavior & Health Education, Department of Kinesiology and Health Education, The University of Texas at Austin, Austin, TX 78712, USA

**Keywords:** gamification, education, behavior change, badges, leaderboard, motivation, meta-analysis

## Abstract

Background: Gamified reward systems, such as providing digital badges earned for specific accomplishments, are related to student engagement in educational settings. The purpose of this study was to conduct a meta-analytic review to quantify the effects of gamified interventions on student behavioral change. Methods: A meta-analysis was performed using the following databases: The Academic Search Complete, Communication & Mass Media Complete, Education Source, ERIC, Library Information Science & Technology Abstracts, and PsycINFO. Inclusion in the review required: (a) peer-reviewed conducted between 2010 and 2019, (b) experimental controlled design, (c) gamification elements, and (d) educational setting. Results: Using a random-effects model, a statistically significant (Cohen’s d (ES) = 0.48, 95% CI = 0.33, 0.62) gamification effect was evidenced by moderate and positive grand effects sizes (ES). Gamification effects were higher with adults in higher education (ES = 0.95) than K-12 students (ES = 0.92). Brief interventions delivered in days or less than 1 week were significantly more effective (ES = 1.57) than interventions lasting up to 20 weeks (ES = 0.30). Interventions incorporating gamification elements across years (ES = −0.20) was adversely associated with behavioral change. Conclusions: Findings suggest that short-term over longer-term gamified interventions might be a promising way to initiate changes in learner’s behaviors and improve learning outcome.

## 1. Introduction

Motivation is a mental process that brings about and maintains goal-oriented actions [1]. It is essential for learning and the acquisition of knowledge [2]. One factor influencing teaching and learning is the increased 24/7 access and reliance on the Internet and mobile devices. Expanded access to the Internet has changed the way we learn [3]. It is not just the expanded access to information, but also interactive social media, streaming videos, enabled online gameplay, and public health information that influence learning [4]. Online learning environments can be enriching because of timely student feedback and the multiplicity of platforms for expression and simulation. Given the paucity of research about gamified learning instructional strategies, the impact of such benefits needs to be quantified [5]. Specifically, online gamified learning as a motivational strategy in education and how gamification is related to student motivation and performance warrants further investigation. Empirical research is essential to determine the possible online gamification effects of badges, leaderboards, wearable devices, and community challenges to increase student motivation.

One common gamification method is the use of achievement badges. Typically, there is no practical value in being awarded a badge; however, attaining a badge creates a sense of satisfaction because receipt of a badge acknowledges progress toward accomplishing the desired outcome. The pursuit of badges is an emotional investment that symbolizes the magnitude of a challenge [5]. Using badges as rewards for achieving goals have a long history. For example, organizations like the Boy Scouts of America and Girl Scouts of America award badges for demonstrating a specific proficiency of a skill (e.g., starting a fire). For adults, airlines award elite status for meeting threshold amounts of travel [6]. The strength and utility of an educational badging system are related to the learning engagement and assessment. The conscious awareness of emotional commitment can enrich the learning experience and help the students see the inherent value of refining or obtaining a new skill. It is believed that the strength and utility of an educational badging system are associated with the context and should be directly aligned with the learning engagement and assessment strategies [7].

Commercially, gamification has been successfully integrated into platforms, especially social ones, to create targeted relationships between the software application and the users to drive viral behaviors that increase popularity [8]. It has been proposed that gamification likely has its place in education to increase student engagement and motivation to achieve learning standards [9]. Its potential benefits may address well-known issues as, e.g., the lack of student motivation due to the limited capacity of interaction with teachers and students [10].

Self-Determination Theory (SDT) is a motivation process with three emotional states of intrinsic motivation, extrinsic motivation, and amotivation [11]. SDT is grounded in three essential human psychological needs: competence, autonomy, and relatedness [12]. Competence, knowing that one was successful, can be enhanced from feedback for success [13]. Autonomy is defined as the degree to which individuals perceive themselves as responsible for the initiation of the behavior. Relatedness is the need to perceive that one can associate with others and with the social world in general [14]. Because gamification includes online badges, ownership, and leaderboard [15], it can be a factor covers competence, autonomy, and relatedness to fulfill intrinsic motivation.

Recent research has tried to find connections between SDT and gamification using meta-analysis. These find shows the overall significant, small positive effects of gamification on cognitive, motivational, and behavioral learning outcomes in a general learning environment [16]. Additionally, another meta-analysis confirms this result that gamification does appear to have a positive and significant small to medium effect on student learning outcomes in educational settings [17].

These studies led researchers to theorize that gamification could also be used in education as a tool to increase students’ engagement and to drive them toward desirable learning behaviors [18]. The potential benefits of gamified learning may address public issues such as the lack of student motivation due to the limited interaction with teachers and students [19].

For this review, gamification was operationalized as any gamelike element applied in a non-game context like a learning environment [20]. Gamification is thought of as both a game element and as the process of creating gameful experiences to increase motivation to sustain desired behaviors [21]. The following gamification elements were examined within the review: badges, leaderboard, points, achievements, levels, story/theme, clear goals, feedback, rewards, progress, and challenge, because these have been linked to increased motivation [9]. Acquiring gamification rewards motivates the learner to participate in the educational environment and activities continuously. The action of earning badges can thus drive the acquisition of knowledge and skill [10].

Accordingly, this study aimed to summarize existing research related to using online gamification platform in education. This research was designed to answer the following research questions: How does gamification influence learners’ motivation (e.g., participation level) and performance (e.g., test score)? Do gamification effects differ across age, length of the program, and type of outcome measure? We believed that this systematic review of the literature would reveal gaps in our understanding of how gamification is being used as an approach to increase motivation. We anticipated that gamification elements would have different degrees of influence on motivation and that such differences would be based on the characteristics of the sample, the chronological age of the participants, and the context or circumstance under which the gamification elements were being applied. Identifying and addressing the gaps in the literature has implications for gamification in an educational setting.

## 2. Materials and Methods

The present systematic review was conducted using the Preferred Reporting Items for Systematic Reviews and Meta-Analyses (PRISMA) [22] to identify the gamification effects on student motivation and performance in education. The number of gamification articles in 2010 (*n* = 63) has exponentially increased (2019, *n* = 1290), thus reflecting its importance as a motivational strategy in interventions.

### 2.1. Procedure

The PRISMA checklist was organized into the procedural steps of identification, screening, eligibility, and included. The PRISMA flowchart displays an overview of the process.

#### 2.1.1. Search Procedure and Selection Criteria

At the outset of the review, each term of interest, “gamification” and “education”, was operationally defined and used as a search term. Related antecedents or words that may have similar meanings were identified and included in the search, such as “online badges”, “leaderboards”, and “motivational affordance” were included in the search filters because it had been identified as keywords in other gamification publications. We excluded “game-based learning” and “serious game” using the game itself rather than gamification that uses the application of game-design elements such as online badges and online leaderboard.

The search syntax was “gamification” AND “education” AND “motivational affordance” AND “online badges” AND “leaderboard” NOT (“game-based learning” AND “serious game” AND “online game”). The term “gamification” first appeared in 2008 [23], but it was not used widely in the research area until 2010 [24]. The search engines of Academic Search Complete, Communication & Mass Media Complete, Education Source, ERIC, Library Information Science & Technology Abstracts, and PsycINFO were used to identify relevant studies published between 2010 and 2019. Reference sections of studies were also examined to identifying additional studies that met the inclusion criteria.

The search findings were screened to eliminate any articles that do not meet the minimum inclusion criteria. For example, non-empirical articles or unpublished dissertations were excluded from the analysis. The following were the inclusion criteria for the review: (a) peer-reviewed, articles published in English, (b) empirical research with a control group, (c) gamification elements rather than on game-based learning or full games, (d) gamification was an independent or exposure variable, and (e) education setting.

**Figure 1:**
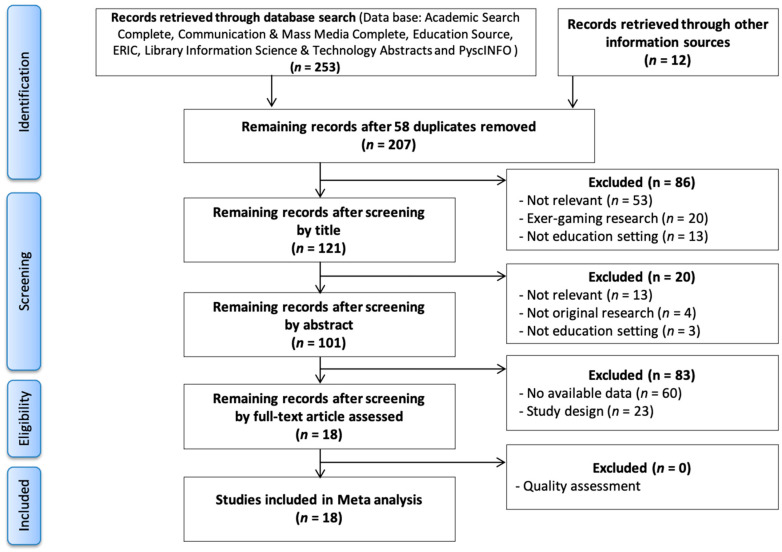
Preferred Reporting Items for Systematic Reviews and Meta-Analyses (PRISMA) flow chart.

Figure 1 displays the steps of identification, screening, eligibility, and inclusion that were carried out. Initially, 253 potential studies on gamification effects on learner outcomes were identified and screened. Fifty-eight duplicate articles were removed, while 12 additional articles were identified from the reference list of the original articles. After screening by the title and authors of the articles, there were another 86 records removed from consideration because 53 abstracts revealed that the article was not relevant to the current study, 20 articles were about exergaming only and did not include the gamification elements of interest in this review, and 13 articles were studies that were not conducted in an educational context. In the screening step, 101 abstracts were read, resulting in the elimination of 20 articles because these were not relevant to this present study. In addition, of the 101 abstracts, 83 potential citations were excluded. Because the articles were not experimentally designed to compare between an experimental and a control group for conducting a meta-analysis.

#### 2.1.2. Assessment of Study Methodological Quality

During the screening step, two reviewers inspected the full text of included studies and independently coded the research methodological to assess its rigor using the Downs and Black checklist [25]. The modified Downs and Black checklist permitted the reviewers to determine the quality of the research across different methodologies and approaches [26,27]. The modified checklist was utilized to analyze the study’s validity and power more explicitly. With a sum score of 28, the higher score indicated a higher level of methodological rigor. For example, if a power or sample size calculation was mentioned, it scored a 1. If the sample size and power calculation were explained and whether the number of participants was mentioned and appropriate for the addressed question, this also earned 1 point. All discrepancies between reviewers were resolved through research team debriefing and consensus. The percentage agreement for two raters was calculated for inter-rater reliability (reporting: 88.8%, external validity: 90.6%, internal validity: 91.4%, and power: 94%). Any study that scored relatively low on methodological quality was not considered for inclusion in the meta-analysis.

#### 2.1.3. Data Extraction and Coding

Five categories of variables were extracted and coded from each of the included studies: (a) study characteristics (study year, author), (b) participant characteristics (age (k-12), college students, and adults), (c) intervention length (less than 1 h, 2–16 weeks, and 1–2 years), (d) gamification type (online badges, leaderboard, levels, progress bar, points, and avatars), and (e) statistical data (control and treatment outcomes). Again, all codes were confirmed using two raters, research team debriefing, and consensus. The percentage agreement for two raters was calculated for inter-rater reliability (study characteristics: 95.5%, participant characteristics: 93.9%, intervention length: 84.8%, gamification type: 81.8%, and statistical data: 93.9%). The primary outcome variables were defined as changes in learner’s test scores and participation levels across three different age groups, intervention length, and measurement (Table 1).

To determine the influence of moderator variables on gamification’s overall effect size (ES) values for learner’s behavioral change, we extracted three variables (age, intervention length, and measurement) from each included study. Age was classified as kindergarten through 12^th^ grade (K-12), college students, and adults. Two studies included K-12, 10 studies included college students, and 6 studies included adults. Intervention length was classified as days, weeks, and years to discover whether variation in the length of the gamification intervention produced differential effects on the learner’s behavioral change. Measurement outcome was categorized as a test score or participation level. When there was insufficient data information to compute an ES, we contacted the corresponding author of each related study via email to obtain means and standard deviation.

### 2.2. Data Analysis

The researcher conducted a meta-analysis on the findings from the systematic review, using the codes from the PRISMA guidelines and the mean difference values from research articles. The mean ES values, along with 95% CIs, were estimated using a random-effects model for all outcomes [3,28,29,30,31,32,33,34,35,36,37,38,39,40,41,42,43,44] displayed in Table 1. To compute ES measures, mean group differences of final test score and participation level between gamification and control group were used. According to Cohen’s [45] definition, ESs were classified as small = 0.2, medium = 0.5, and large = 0.8.

The heterogeneity of weighted mean ES was examined through moderator analysis using Cochran’s Q statistics (Q) [46]. When the Q statistic was significant (*p* < 0.05), it indicated heterogeneity of effects, so we performed additional analyses to examine the effect of each moderator. The amount of potential publication bias was also analyzed via visual inspection of a funnel plot and Egger’s test of the regression intercept and used this as a final determination for inclusion. All statistical data analyses were conducted using Comprehensive Meta-Analysis version 2 software program that provides a complete set of analytical ways to conduct a meta-analysis [47].

## 3. Results

A total of 253 publications from 5 databases and 12 potentially relevant studies from the reference lists of the included articles were considered for further review. After a preliminary review, 58 studies were eliminated due to their inability to meet criteria and duplication. We retrieved information from the remaining 207 studies first by title and abstract, then by full-text, but upon screening these studies did not meet the inclusion criteria (i.e., 86 and 20 studies by step). Full texts of the remaining 83 studies were reviewed for a detailed assessment. A total of 18 studies provided sufficient data to compute an ES and were included in this analysis. The methodological quality of the included studies was fair (mean ± standard deviation (SD) 17.11 ± 1.37, ranging from 15 to 20, considering the maximum score of 28) according to the previous research [27]: excellent [26,27,28], good [20,21,22,23,24,25], fair [15,16,17,18,19], and poor (<15). No studies had quality scores outside two standard deviations of the mean. Average scores for each measurement domain were: (a) reporting (9.00 of 11), (b) external validity (0.94 of 3), (c) internal validity (7.17 of 13), and (d) power: (0 of 1).

### 3.1. Overall ES

**Figure 2:**
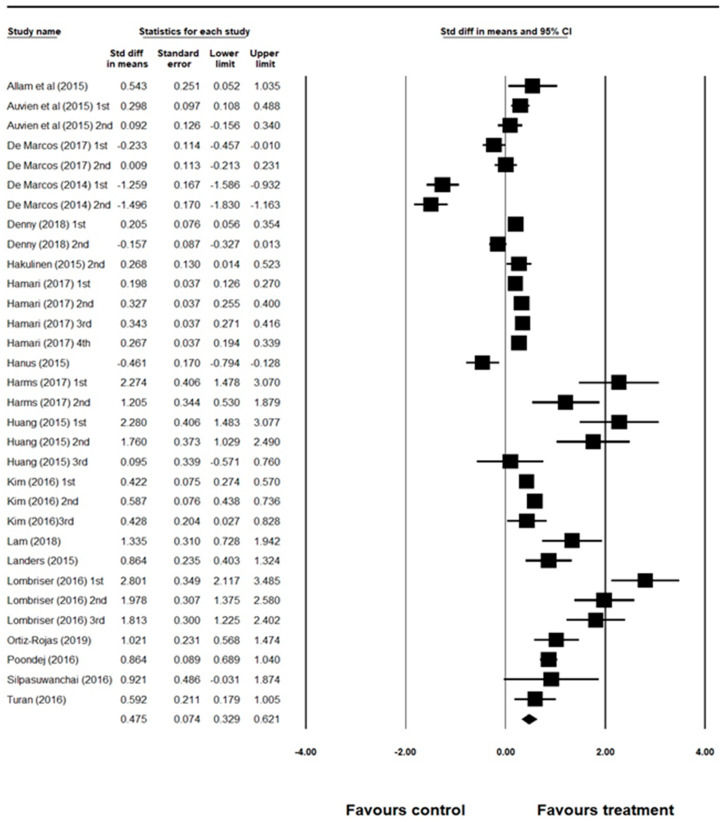
Standardized mean difference effect sizes, 95% CI and a forest plot.

The weighted mean ES values, 95% confidence interval (CI), and a forest plot are provided in Figure 2. Overall, 32 ESs were calculated from the 18 studies. The results from ES calculations indicated that the treatment (gamification) effect was statistically significant (Cohen’s d (ES) = 0.48, 95% CI = 0.33, 0.62), moderate, and positive mean ES, using a random-effects model. This finding indicated that gamification is a useful motivational tool to increase learner’s behavioral outcomes.

### 3.2. Moderator Analysis

Moderator analyses were performed to examine the effect of age (i.e., K-12, college students, and adults) and intervention length (i.e., days, weeks, and years) as independent variables and measurement (i.e., test score and participation level) as dependent variable on overall weighted mean ES. Table 1 indicates the results of moderator analysis, which provides ES, 95% CI, and Cochran’s Q statistic for each moderator variable (Table 2.) Cochran’s Q test is a nonparametric statistical test that assesses whether the treatments have the same effects among groups [48].

The results of moderator analysis indicate that the Q statistic for the age (K-12, college students, and adults) and intervention length (less than 1 h, 2–16 weeks, and 1–2 years) were statistically significant. The Q statistic for age, Q between (Qb) = 26.27, df = 2, *p* < 0.01, explained the heterogeneity of ESs. The adults intervention (ES = 0.95, 95% CI = 0.70, 1.12) appeared to be more effective than K-12 (ES = 0.92, 95% CI = 0.29, 1.55) and college students intervention (ES = 0.15, 95% CI = −0.04, 0.35). The Q statistic for the intervention length also indicated that less than 1-h intervention (ES = 1.57, 95% CI = 1.25, 1.90) appeared to be more effective than 2–16 weeks (ES = 0.39, 95% CI = 0.21, 0.57) and 1–2 years (ES = −0.20, 95% CI = 0.40, 0.77) intervention groups in behavioral change.

### 3.3. Publication Bias

**Figure 3:**
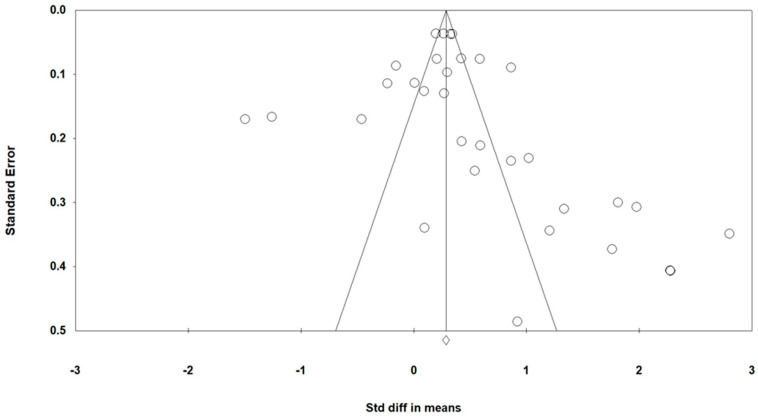
Funnel plot of all 32 effects from treatment and control samples.

Meta-analysis results may not describe the population of interest due to publication bias, which happens when studies with statistically significant results tend to be published than studies with statistically nonsignificant results. The funnel plot was created to assess the presence of publication bias (Figure 3). When publication bias has occurred, sections of the funnel may be missing, or the plot may become very asymmetrical [49]. The plot appears to be more positive effects than negative ones; however, Egger’s test of regression intercept was 1.46 (*p* = 0.22), which indicates that the potential for publication bias was minimized across the studies.

## 4. Discussion

We examined the relationship between gamification, as specific elements and as a process, and a behavioral change in education settings using the meta-analysis technique. The results show that the gamification strategy has a moderate, positive effect on engagement behaviors and test scores. This study also examined if age, intervention length, and measurement type influence the effectiveness of the gamification intervention. The findings in this study are based on 32 data sets from 18 experimental design studies. We realize that this body of literature is growing exponentially and that even though this is an adequate volume of experimental data to conduct these analyses, we acknowledge that additional experimental research was conducted concurrently and could not be included in this review. Consequently, this is viewed as one of the leading researches [16,17] to quantify and qualify gamification effects in educational settings using meta-analysis.

### 4.1. Developmental Stage and Gamified Interventions

In the present study, participants were categorized into three different age groups (K-12, college students, and adult non-student), where each group has a different ratio of the amount and estimated their ESs. There is a significant difference in ESs between the three age groups in this study. The gamified intervention effects were most significant for older adults compared to those of K-12 and college students. This result indicates that there might be a possibility that younger age people and older people were more interested in gamified factors in education than college students’ age groups. Contextual and developmental factors may have influenced the effectiveness of interventions focused on these portions of the lifespan, but such an analysis was beyond the scope of this review.

Older adults demonstrated the highest engagement compared to college students and K-12 students. Wang and colleagues [50] found that older users are more easily influenced by social modeling than younger adults. Leaderboards are a mechanism of social comparison. One’s place or absence on the leaderboard can have differential effects related to mastery and ego-oriented motivation. The more inferior effect of social influence on the younger generation, maybe because they have been exposed to a gamified strategy at a younger age [51]. This result was contrary to previous research that the influences of age in technology adoption and usage have designated that younger technology users value the technology’s usefulness more than older [52]. As a transitional stage, it can be assumed that young adults possibly lost their interest in gamified features that they held when they are young. Older participants are attracted to the gamified elements because they emphasize ease of achieving goals by reflecting progress [53]. The novelty of the gamified elements is likely driving the effects [54]. Over time, college students’ age and developmental stage have shifted away from exclusively emerging adulthood to representing a diversity of developmental stages across the lifespan. Instructors in higher education need to be aware that gamification could be useful for more non-traditional and older adults. These finding warrants further investigation to understand the effects of a gamification strategy in education by age groups.

### 4.2. Length of Gamified Interventions

Given the data reviewed in this study, there is an optimal length of gamified interventions. People often prefer short-term rewards rather than long-term rewards in modern life [55], and this cognitive inclination is called hyperbolic discounting [56]. Gamified interventions lasting days were significantly more impactful than those lasting 1–2 years. This finding provides a practical implementation that learners possibly have more motivation for learning or participation in intensive, short-term scenarios than in extended education settings [57]. It is recommended that we investigate students’ needs and motivations to carefully plan and examine the rewarding design, considering timing and duration to adequately address the motivational affordances that create compelling socially gamified learning experiences [58]. Further, the timing of new challenges (e.g., gamified as levels or events) and how long it takes someone to earn a new badge) need to be investigated in relation to the developmental stage, context, and intervention length, as more data are needed to support the reliability of this assertion.

### 4.3. Behavioral Change and Learning Outcome

This study indicated ESs for different outcome measurements to examine if gamification affects differently on outcome measurements such as participation level and test score. These results have shown that there is no significant difference in outcome measurement. However, participation level (ES = 0.60, 95% CI = 0.40, 0.77) had higher effect size than those of test score (ES = 0.30, 95% CI = 0.03, 0.18). This result suggests that gamification has more effect on a learner’s participation level than a test score. Increased learning time, such as participation level, may develop learning skills and academic achievement [59]. Subsequently, it is expected that educators improve learners’ participation levels (e.g., learning time) using gamification strategy, impacting learning outcomes [60].

### 4.4. Study Delimitations and Limitations

These meta-analysis findings are significant because gamification is an emerging and growing issue in education [58]. Although this direct mechanism has not yet been adequately investigated in educational settings, it has been confirmed that the gamification strategy increased the learner’s behavioral change, including test score and participation level. Therefore, although limited in scope, experimental investigation supports the hypothesis that gamification motivates learners’ positive reviewers’ change. This study’s main strength was the deliberation of gamification as a motivation strategy for learners’ positive behavioral change and learning outcome in education. This study has also shown the moderating effect of age group, intervention length, and measurement type, which could help plan gamification-based education programs.

Another distinctive characteristic of this study was the methodological quality. The average Downs and Black Scale total score was fair (mean ± standard deviation (SD) 17.11 ± 1.37, ranging from 15 to 20, considering the maximum score of 28). Consideration of study quality is a unique feature of this gamification study. The meta-analysis has shown that gamification affects learners’ positive behavioral change, but there are limitations explaining its impact on learners’ behavior. Most of the studies used diverse gamification elements, including online badges and leaderboards only, and some combined with other sources such as progress bar or rewards points. Future research should aim to use objective measurable treatments, e.g., online badges and leaderboards only.

All studies in this meta-analysis were quasi-experimental instead of randomized control experimental design because there are limitations for conducting randomized sampling in an educational setting. Although the overall ES of our study demonstrated that the gamification strategy has moderate effects on the learner’s behavioral change (ES = 0.48), the results should be interpreted with caution due to the lack of casual outcomes. Based on the funnel plot, studies indicating that the publication bias was minimized across the studies.

### 4.5. Implications for Gamified Educational Learning

The present study examined the overall ES of gamification on learners’ behavioral change. The evidence suggests that gamification has a moderate and positive effect on learner’s behavioral change in gamified intervention studies. The results indicated that gamification impacts are similar across all types of outcome measurements. However, the different age groups and intervention lengths have a diverse effect on the learner’s behavioral change. The gamification effect on college students is relatively lower than those of school ages students and adults. However, a fundamental question driving every meta-analytic research is generalizability [61]. Therefore, it should be careful to conclude that college students are not highly motivated by the gamified teaching method. However, this result can imply that educators should be cautious in designing game mechanics at college-level programs.

Contrary to previous findings suggesting that gamified interventions of 20 weeks offering badges to children who participated in physical activity breaks in the classroom significantly increased children participation [7], the summary of research, in comparison to college students and older adults, did not produce the same degree of behavior change. Short-term gamification intervention with K-12 students in their participation level has shown the comparatively most significant effect of learners’ behavioral change, and so we would advocate for its continuation, but recommend that intervention length, gamification elements and its timing, and developmental stage be thoughtfully mapped onto the outcome variable. It is based on the same idea that gamified or gameful motivational tools are most beneficial to younger ages [62]. The evidence presented here can help design optimal gamification interventions that maximize increases in K-12 learners’ positive behavioral change.

## 5. Conclusions

The variations in gamification effect across different intervention length and the significant impact of moderators suggest that different conditions influence gamification’s effects on behavior change. The present study results can provide useful information for educators to use gamification as an effective intervention strategy. Additional research is also needed to use more gamification types (i.e., online badge, leaderboard, progress bar, points, and avatar) and diverse programs in K-12 educational settings.

## Figures and Tables

**Table 1:** Characteristics of gamification intervention studies included in the meta-analysis.

Study (Year)	Treatment *n*	Control *n*	Age Group	Intervention Length	Measurement Outcome	Gamification Affordance	Education Field
Allam et al. (2015) [28]	28	40	Adult	Weeks	Test score	Badges, leaderboard, points	Medical education
Auvien et al. (2015) [3]	215	215	CS	Weeks	Test score	Badges, points	Computer science
	254	254	CS	Weeks	Test score		
De Marcos et al. (2017) [29]	175	139	CS	Weeks	Test score	Badges, leaderboard, points, challenge, goals, levels, peer assessment	Computer science
	177	139	CS	Weeks	PL		
De Marcos et al. (2014) [30]	106	72	CS	Years	Test score	Badges, leaderboard, points, progress bar	Computer science
	112	72	CS	Years	PL		
Denny et al. (2018) [31]	702	702	CS	Weeks	Test score	Badges, points	Online education
	521	180	CS	Weeks	Test score		
Hakulinen et al. (2015) [32]	86	195	CS	Weeks	PL	Badges, leaderboard, points	Computer science
	86	195	CS	Weeks	PL		
Hamari (2017) [33]	1579	1401	Adult	Years	PL	Badges	Online trading activity
	1579	1401	Adult	Years	PL		
	1579	1401	Adult	Years	PL		
	1579	1401	Adult	Years	PL		
Hanus & Fox (2015) [34]	71	71	CS	Weeks	PL	Badges, leaderboard	Communication
Harms et al. (2017) [35]	21	19	Adult	Hours	PL	Badges, avatar, progress bar	Physical activity
	21	19	Adult	Hours	Test score		
Huang and Hew (2015) [36]	21	19	CS	Weeks	PL	Badges, leaderboard, points, progress bar	General Education
	21	19	CS	Weeks	PL		
	19	16	CS	Weeks	Test score		
Kim et al. (2016) [37]	448	299	CS	Weeks	PL	Badges, leaderboard	Engineering
	448	299	CS	Weeks	PL		
	51	47	CS	Weeks	PL		
Lam et al. (2018) [38]	22	30	K-12	Weeks	PL	Leaderboard, points,	ESL writing
Landers et al. (2015) [39]	33	49	Adult	Hours	Test score	Leaderboard, points,	Brainstorming
Lombriser et al. (2016) [40]	51	21	Adult	Hours	PL	Badges, leaderboard, points, level, challenges, avatar, progress bar, storytelling, prize	Engineering
	51	21	Adult	Hours	Test score		
	51	21	Adult	Hours	PL		
Ortiz-Rojas et al. (2019) [41]	55	34	Adult	Hours	Test score	Leaderboard	Computer programing
Poondej et al. (2016) [42]	273	273	CS	Weeks	PL	Badges, leaderboard, point, progress bar	Information literacy skills
Silpasuwanchai et al. (2016) [43]	19	6	CS	Hours	PL	Badges, leaderboard, points	Memory
Turan et al. (2016) [44]	46	48	K-12	Weeks	Test score	Badges, leaderboard, points	Technology software

CS = college students; K-12 = kindergarten through 12th grade; ESL = English as a Second language; PL = participation level; Hours = less than 1 h; Weeks = 2–16 weeks; Years = 1–2 years.

**Table 2:** Effect sizes by moderator variables in the meta-analysis.

Moderator Variables		*n*	ES	95% CI		Qb
				Lower	Upper	
Age	K-12	146	0.92	0.29	1.55	
	College students	5780	0.15	−0.04	0.35	26.27 **
	Adults	12,455	0.95	0.70	1.12	
Intervention length	Days	492	1.57	1.25	1.90	
	Weeks	12,282	0.39	0.21	0.57	67.20 **
	Years	18,381	−0.20	−0.47	0.09	
Measurement	Test score	3059	0.30	0.03	0.18	3.38
	Participation level	15,322	0.60	0.40	0.77	

** *p* < 0.01.

## Data Availability

Not applicable.
